# Air Quality Improvement Is Associated With Decreasing Depressive Symptoms in Older Women

**DOI:** 10.1093/geroni/igab046.1317

**Published:** 2021-12-17

**Authors:** Andrew Petkus, Xinhui Wang, Diana Younan, Daniel Beavers, Mark Espeland, Joshua Millstein, Margaret Gatz, Jiu-Chiuan Chen

**Affiliations:** 1 University of Southern California, LOS ANGELES, California, United States; 2 University of Southern California, Los Angeles, California, United States; 3 Wake Forest School of Medicine, Winston-Salem, North Carolina, United States

## Abstract

Air pollution exposure is an environmental risk factor in brain aging and may also be associated with late-life depressive symptoms (DS). It is unknown if air quality (AQ) improvement is associated with reductions in DS in later life. Longitudinal data from 917 cognitively intact women with no prior history of depression (baseline age 66.4 ± 1.6 years old) participating in the Women’s Health Initiative Memory Study of Younger Women (WHIMSY; 2008-2016) were analyzed to examine whether AQ improvement over the 5-years prior to WHIMSY baseline was associated with trajectories of DS (measured by 15-item Geriatric Depression Scale). Annual PM2.5 (fine particulate matter of aerodynamic diameter <2.5) and NO2 were estimated at the participants’ residence using regionalized universal kriging models. Estimates were aggregated to the 3-year average at 5 years (remote) and immediately (recent) before WHIMSY baseline. Associations between AQ improvement (difference between remote to recent exposure) and trajectories of DS were estimated using linear mixed effect models, adjusting for sociodemographic, lifestyle, and clinical covariates. AQ improved prior to baseline (PM2.5: 1.62 ± 1.45 μg/m3 and NO2: 3.70 ± 2.81 ppb). Women residing in locations with greater improvement in NO2 (per IQR = 4.34 ppb) or PM2.5 (per IQR = 2.30 μg/m3) reported significant annual reductions in DS (βNO2=3.1%, p=.046; βPM25=1.6%, p=.046), similar to the effect of engaging in moderate to vigorous physical activity four times or more a week. These findings suggest that improving air quality may reduce depressive symptoms in older women.

